# Myosin folding boosts solubility in cardiac muscle sarcomeres

**DOI:** 10.1172/jci.insight.178131

**Published:** 2024-03-14

**Authors:** Colleen M. Kelly, Jody L. Martin, Michael J. Previs

**Affiliations:** 1 Molecular Physiology and Biophysics Department, Larner College of Medicine, University of Vermont, Burlington, Vermont, USA.; 2 Department of Pharmacology, University of California, Davis, Davis, California, USA.

**Keywords:** Cardiology, Muscle biology, Cytoskeleton, Muscle

## Abstract

The polymerization of myosin molecules into thick filaments in muscle sarcomeres is essential for cardiac contractility, with the attenuation of interactions between the heads of myosin molecules within the filaments being proposed to result in hypercontractility, as observed in hypertrophic cardiomyopathy (HCM). However, experimental evidence demonstrates that the structure of these giant macromolecular complexes is highly dynamic, with molecules exchanging between the filaments and a pool of soluble molecules on the minute timescale. Therefore, we sought to test the hypothesis that the enhancement of interactions between the heads of myosin molecules within thick filaments limits the mobility of myosin by taking advantage of mavacamten, a small molecule approved for the treatment of HCM. Myosin molecules were labeled in vivo with a green fluorescent protein (GFP) and imaged in intact hearts using multiphoton microscopy. Treatment of the intact hearts with mavacamten resulted in an unexpected > 5-fold enhancement in GFP-myosin mobility within the sarcomere. In vitro biochemical assays suggested that mavacamten enhanced the mobility of GFP-myosin by increasing the solubility of myosin molecules, through the stabilization of a compact/folded conformation of the molecules, once disassociated from the thick filaments. These findings provide alternative insight into the mechanisms by which molecules exchange into and out of thick filaments and have implications for how mavacamten may affect cardiac contractility.

## Abstract 2

The small molecule mavacamten regulates the folding of myosin molecules to enhance their mobility within cardiac muscle and may be a therapeutic opportunity for hypertrophic cardiomyopathy.

## Graphical abstract

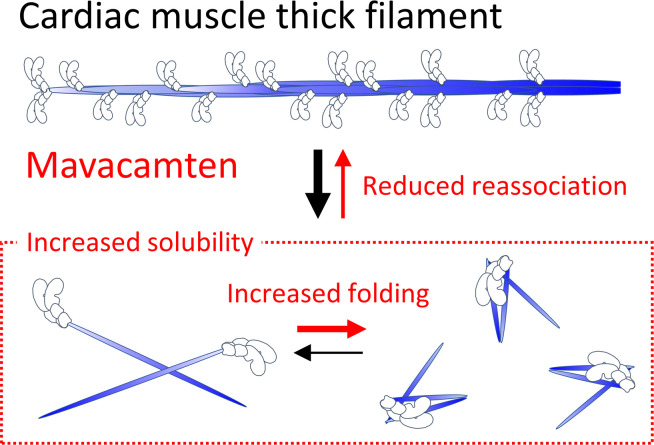

## Introduction

**Figure 1:**
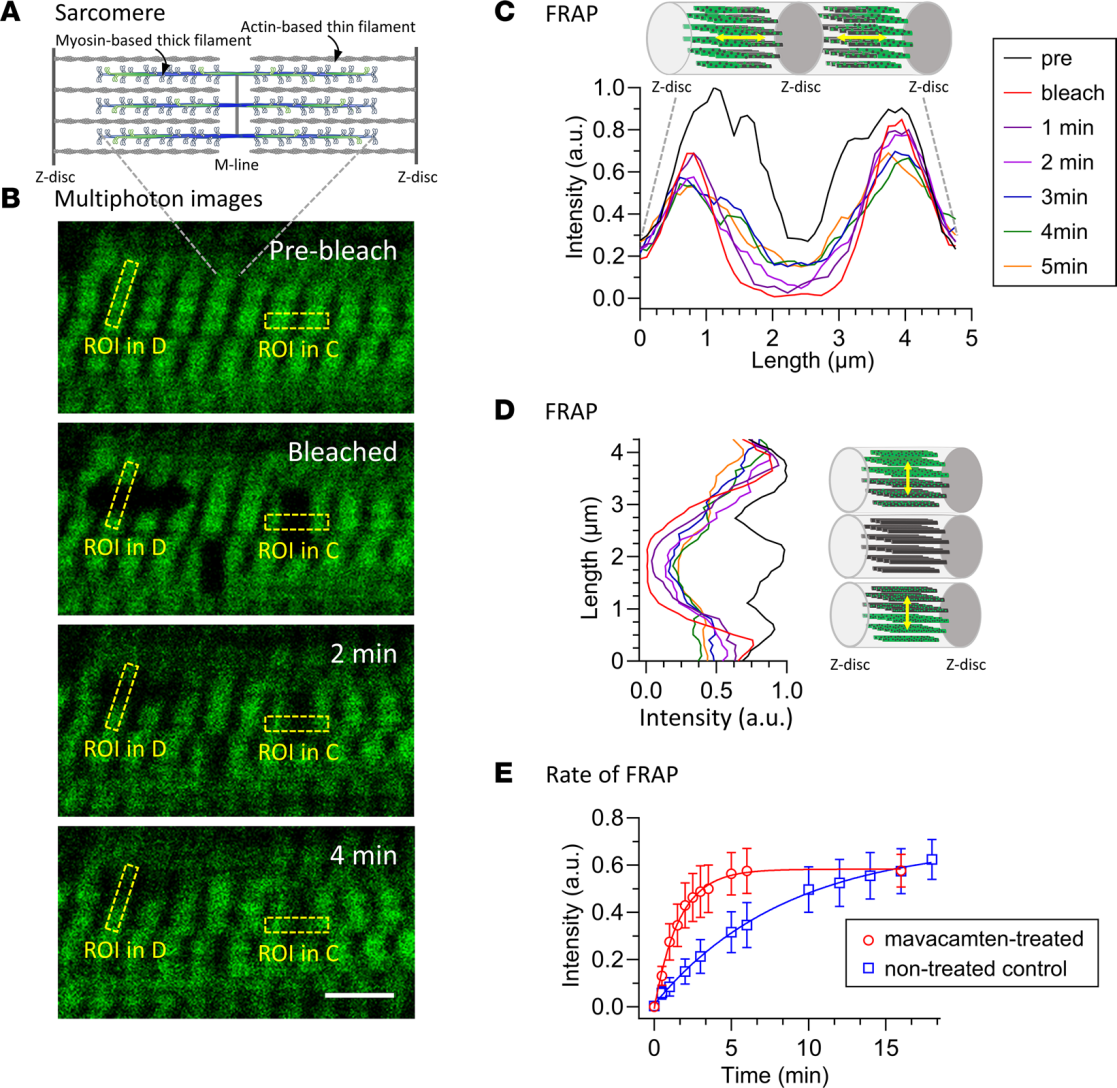
Quantification of the effect of mavacamten on MYL2-GFP mobility within single sarcomeres. (**A**) Illustrative representation of a single sarcomere containing GFP-labeled myosin molecules (green). (**B**) Series of multiphoton images of MYL2-GFP fluorescence within a cardiac muscle cell, in intact myocardium treated with 10 μM mavacamten. Scale bar: 5 μm. (**C** and **D**) Fluorescence intensity distribution profiles along the long axes of the ROIs shown in **B**, and illustrative representations of the arrangement of sarcomeres. The change in fluorescence intensity within 2 sarcomeres connected in series by a Z line within a single myofibril (**C**) and 3 separate sarcomeres within parallel myofibrils (**D**). (**E**) The average fluorescence recovery (± SEM) after photobleaching a portion of single sarcomeres plotted versus time. These averages were generated from 30 sarcomeres within 3 hearts treated with 10 μM mavacamten and 30 sarcomeres within 5 nontreated control hearts. Statistical significance (*P* < 0.001) was determined by extra sum-of-squares F test.

The continuous beating of the heart results from the antiparallel sliding of actin-based thin filaments along myosin-based thick filaments, organized within muscle sarcomeres (Figure 1A). Myosin is a 520 kDa hexamer composed of 2 heavy chains and 4 light chains. The C-terminal and central regions of the 2 heavy chains dimerize to form a long coiled-coil, which serves as the tail of each molecule. This tail allows for intermolecular electrostatic interactions between ~300 myosin molecules (1–3), myosin-binding protein C, and titin (4, 5), to form each ~1.65 μm bipolar thick filament (6). The N-terminal region of each heavy chain forms a globular head and a lever arm that is decorated with an alkali/essential light chain and a regulatory light chain. This globular head binds to actin and utilizes energy captured from the hydrolysis of adenosine triphosphate (ATP) to swing the lever arm (7, 8). This results in the sliding of the actin filaments toward the center of the thick filament, shortening the sarcomere and, thus, cellular length during each cardiac muscle contraction.

In healthy hearts, the binding of myosin to actin is regulated by both the availability of myosin heads protruding from the thick filament backbone and their access to binding sites on the actin filament (9, 10). The availability of the myosin heads is postulated to be modulated by both biochemical and structural mechanisms. Myosin molecules have been demonstrated to exist in an equilibrium between a disordered relaxed (DRX) and a super-relaxed (SRX) state, which are defined by a 10-fold difference in the rate of ATP turnover (11–16). The SRX state has been proposed to result from an autoinhibited structural state within the thick filament, termed the interacting heads motif (IHM). This structural state has been observed from preparations of single myosin molecules (17–21), thick filaments (4, 22–25), and in intact sarcomeres (5) using electron microscopy. However, the mechanistic links between the biochemical and structural states are unresolved (26–29).

Regardless of the mechanistic detail, disruptions in the SRX state and/or the IHM (16, 30, 31) are proposed to underly the development of hypertrophic cardiomyopathy (HCM), which affects ~1 in 500 individuals (32). HCM is most often associated with genetic mutations in myosin itself (33–35), or myosin-binding protein C (36, 37), being localized within the thick filament C-zone. These HCM-causing mutations are proposed to alter cardiac contractile parameters by effectively increasing the availability of the myosin heads to bind to the actin filaments (16, 30, 31). This increase in myosin availability results in hypercontractility and impaired relaxation of the left ventricle (16, 30, 31), which is accompanied by morphological thickening of the ventricle walls and decreased blood volume (32, 38).

A pivotal advancement in the treatment of HCM included the discovery and FDA approval of mavacamten, a small molecule inhibitor of myosin, which reduces left ventricular outflow tract (LVOT) obstruction and ventricular hypertrophy to improve patient symptoms (39, 40). Mavacamten has been demonstrated to both enhance the SRX state of myosin in biochemical assays (14, 15, 26, 27) and to fold the globular heads into a compact conformation, being similar to the IHM observed on the backbone of the thick filament (15, 41). Thus, the effect of mavacamten on the biochemical (SRX) and structural (IHM-like) states of myosin is postulated to reduce the availability of myosin heads to bind to actin and offset the defects in the contractile parameters that result in HCM.

Due to the clinical significance of HCM, much emphasis has been placed on generating high-resolution structures of myosin and/or myosin-binding protein C within the thick filament, using electron microscopy (4, 5, 17, 23, 25). However, we have recently demonstrated that, even under normal conditions, the structure of the sarcomere appears highly dynamic in vivo, to presumably allow for the replacement of the contractile proteins through synthesis and degradation (42, 43). Specifically, proteomic data demonstrate that molecules are stochastically replaced from within thick filaments in adult mouse hearts (44). Complementary biophysical data demonstrate that this replacement is facilitated by the localization of myosin molecules being highly mobile within the sarcomere (45). These biophysical data suggest that the sarcomere is designed in such a way as to allow for the dissociation of molecules from the thick filaments, diffusion of the molecules in the space between filaments, and reincorporation of the molecules within the same or a neighboring thick filament on the minute timescale (45). While this continual exchange between the filaments and a dynamic pool of soluble monomers provides a possible explanation for how proteins are stochastically replaced in healthy hearts in vivo, questions remain as to whether this exchange is altered in disease states or can be tuned pharmacologically.

Based on our working knowledge of mavacamten, we hypothesized that treatment of heart preparations with the small molecule would promote the stabilization of an IHM-like structure of myosin molecules within the thick filament and increase intramolecular contacts to limit myosin mobility within the sarcomere. We tested this hypothesis by replacing a fraction of the endogenous myosin regulatory light chain 2 (MYL2) in adult mouse hearts, with MYL2 having green fluorescent protein (GFP) fused to its C-terminus (GFP-labeled MYL2 [MYL2-GFP]). We then quantified the mobility of the MYL2-GFP within single sarcomeres in mavacamten-treated intact heart preparations using fluorescence recovery after photobleaching (FRAP) with multiphoton microscopy. Unexpectedly, we quantified a > 5-fold enhancement in myosin mobility in the presence of mavacamten when compared with that in nontreated control hearts. To examine the mechanisms governing the enhanced mobility, we used a combination of ex vivo extraction, in vitro polymerization, and in vitro microfluidic diffusional sizing (MDS) assays. We demonstrate that mavacamten enhanced the mobility of myosin by increasing its solubility through the stabilization of a compact/folded state of myosin molecules, once they were disassociated from the thick filaments. These results provide mechanistic knowledge into the factors that regulate myosin mobility within the sarcomere and have implications on our understanding of how mavacamten may affect cardiac muscle contractility in the human myocardium.

## Results

### AAV-induced expression of MYL2-GFP resulted in the partial replacement of endogenous MYL2 within cardiac thick filaments.

An adeno-associated virus (AAV) was designed using the cardiac-specific TnT promoter to express MYL2 with GFP fused to its C-terminus (MYL2-GFP) in mouse heart ventricles. Adult male FVBWT mice, being 3–6 months of age, were transduced with the AAV, and the mice were allowed to turn over their endogenous proteins for 12 ± 2 days. We previously demonstrated that the half-life of MYL2 was 10.8 days within mice of similar age (44), and 22% ± 7% of the endogenous MYL2 was replaced with MYL2-GFP by transduction with the AAV using this approach (45). We also demonstrated the MYL2-GFP was properly localized to the heads of the myosin molecules within thick filaments, and its presence did not affect cardiac mass or the ability of myosin to slide actin filaments in an in vitro motility assay (45).

### Mavacamten enhanced the rate of MYL2-GFP mobility with sarcomeres.

To test whether mavacamten affects the mobility of the MYL2-GFP within intact mouse hearts, MYL2-GFP was imaged in ex vivo heart preparations, and fluorescence recovery was quantified after photobleaching a fraction of the MYL2-GFP molecules within single sarcomeres. AAV-transduced mice were euthanized, and their hearts were splayed open and pinned to a Sylgard-treated dish to expose the papillary muscle. The hearts were immediately covered in Tyrode’s solution with 10 μM mavacamten (*n* = 3). To define the localization of the MYL2-GFP, 9 different cells in the papillary muscle within the 3 different heart preparations were imaged at room temperature (22°C ± 2°C) with multiphoton microscopy (Figure 1B). The MYL2-GFP molecules in 16 random, < 10 μm^3^ subcellular volumes were photobleached using a higher-intensity excitation (Figure 1B, bleached). Images were recorded for up to 30 minutes to allow for quantification of FRAP within 30 different sarcomeres (Figure 1, B–E, and Supplemental Video 1; supplemental material available online with this article; https://doi.org/10.1172/jci.insight.178131DS1). These data were compared with those collected from 30 sarcomeres, within 11 cells, from 5 different heart preparations, which were not incubated in mavacamten (Figure 1E).

Striations in fluorescence, being ~1.6 μm in width at half-maximal intensity, were observed within the cells prior to photobleaching (Figure 1, B and C). The striations were due to the localization of the MYL2-GFP within thick filaments organized in sarcomeres connected in series from end to end through their *Z* discs (Figure 1C). Minima were observed along the radial axis of these striations (Figure 1D) due to the organization of the sarcomeres within ~1–2 μm wide myofibrils, as previously demonstrated (45). Fluorescence intensity distribution profiles were generated in regions of interest (ROIs) (Figure 1B) along either the longitudinal (Figure 1C) or radial lengths (Figure 1D) of these sarcomeres to quantify the fluorescence intensity of the MYL2-GFP within single sarcomeres.

The fluorescence intensity distribution profile in Figure 1C resulted from fluorescence within 2 sarcomeres, arranged in series, within a single myofibril. Photobleaching ablated the fluorescence intensity from the M line within each sarcomere (Figure 1A) to the Z line shared between the 2 sarcomeres. FRAP was characterized by an increase in fluorescence intensity within the photobleached region and reciprocal reduction in the fluorescence intensity in the nonphotobleached region of each sarcomere. This relationship demonstrated that FRAP resulted from the exchange of the photobleached MYL2-GFP molecules, with fluorescent MYL2-GFP molecules preexisting in the nonphotobleached region of the sarcomere.

The fluorescence intensity distribution profile in Figure 1D resulted from fluorescence from *Z* disc to *Z* disc within 3 sarcomeres arranged in parallel myofibrils. Photobleaching ablated the fluorescence intensity from *Z* disc to *Z* disc from the sarcomere in the middle and half of the fluorescence intensity within its neighbors (Figure 1D). FRAP was characterized by a reduction in the fluorescence intensity in the nonphotobleached portion of each sarcomere and the redistribution of the fluorescence toward the bleached regions within the images. Although the fluorescence intensity appears to gradually increase in the fully bleached sarcomere, this is due to the large point spread function of the fluorescent MYL2-GFP molecules being redistributed in the neighboring, partially photobleached sarcomeres.

To quantify the rate of FRAP, similar analyses were performed to those shown in Figure 1, C and D, for all 30 sarcomeres within 3 heart preparations incubated in Tyrode’s solution containing 10 μM mavacamten (Figure 1E). The data were well fitted (*R*^2^ = 0.991) with a single exponential with a rate constant of 0.65 (±0.52–0.81) min^–1^, half-life of 1.1 (±0.86–1.4) minutes, and plateau of 0.59 (±0.56–0.62). The *R*^2^ did not improve when fitting with multiple exponentials. These data were compared with those collected from the 30 sarcomeres within 5 heart preparations incubated in Tyrode’s solution lacking mavacamten (Figure 1E). These data were also well fitted (*R*^2^ = 0.998) to a single exponential but the rate constant of 0.12 (±0.10–0.13) min^–1^ and half-life of 5.9 (±5.2–6.9) were different (*P* < 0.001) from that quantified in the presence of 10 μM mavacamten, as determined by an extra sum-of-squares F test. The differences in rate constants and half-lives demonstrate that mavacamten increased the rate of myosin mobility within the sarcomere.

### The abundance of soluble MYL2 extracted from intact muscle was tightly coupled to the abundance of the heavy chain in the presence of mavacamten.

**Figure 2:**
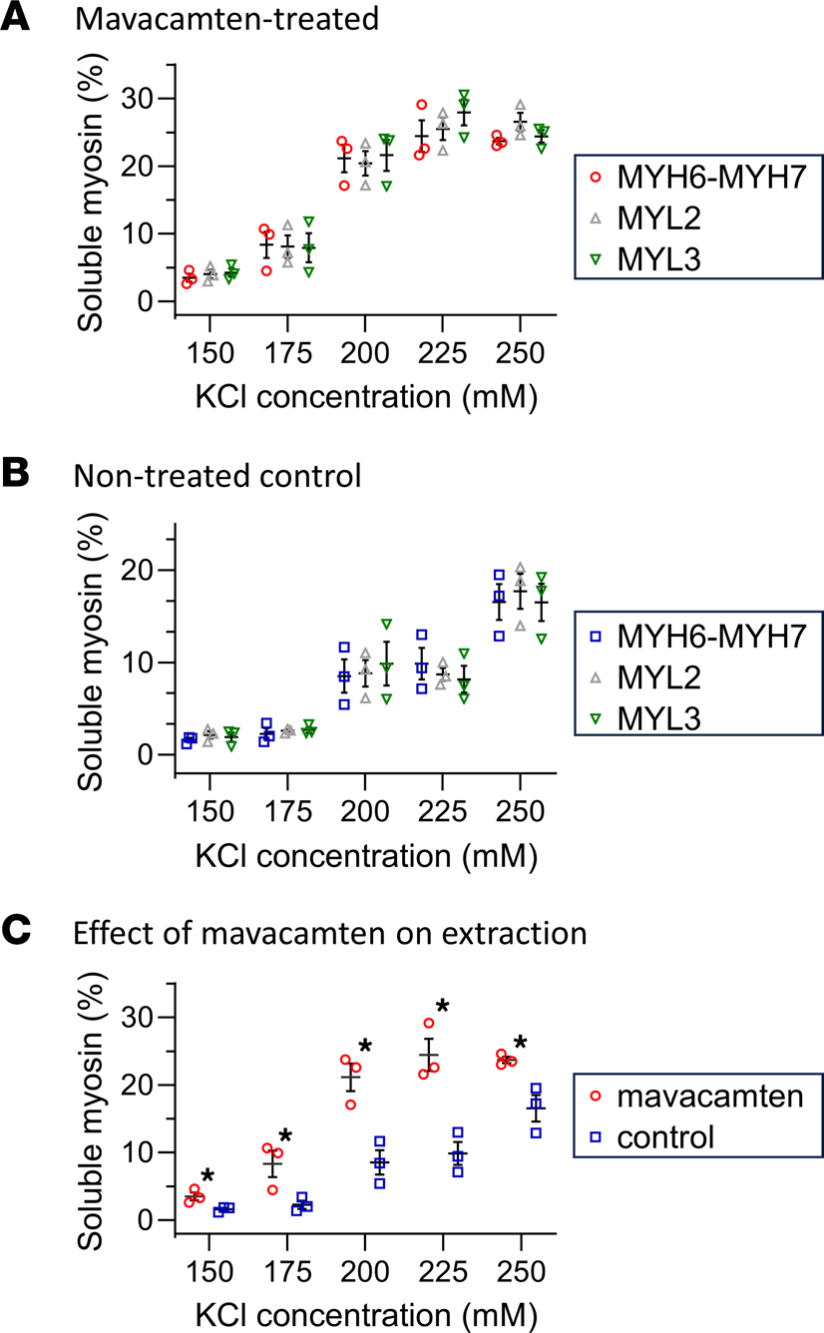
Quantification of the effect of mavacamten on the extraction of myosin from intact cardiac muscle at room temperature (22°C). (**A** and **B**) The average abundances (± SEM) of soluble myosin heavy chain 6 and 7 (MYH6-MYH7), MYL2, and myosin light chain 3 (MYL3) in KCl extraction solution with (*n* = 3) (**A**) and without (**B**) 10 μM mavacamten (*n* = 3). (**C**) The average abundance (± SEM) of myosin in the KCl extraction solution with and without 10 μM mavacamten (*n* = 3). All experiments were performed in the presence of 0.7 mM ATP. *P < 0.05 as determined by unpaired, 2-tailed *t* test.

MYL2-GFP mobility requires the release of molecules from the thick filament, diffusion of these molecules in the space between filaments, and reincorporation of the molecules into a distal location of the same or a neighboring thick filament. To determine whether mavacamten enhanced MYL2-GFP mobility through the selective release of MYL2 from myosin heavy chain (MHC), the abundances of MYH6-MYH7, MYL2, and MYL3, the ventricular alkali isoform extracted from perforated cardiac muscle were determined over a range of ionic conditions. Small, ~1 mg pieces of WT ventricular tissue were triturated for 30 seconds in potassium chloride (KCl) solution containing 10 μM mavacamten, and 0.1% Triton X-100 detergent to solubilize the plasma membrane. The samples were incubated at room temperature (22°C ± 2°C) for 30 minutes to allow the soluble proteins to equilibrate with the solution. The soluble proteins were removed, and the abundances of MYH6-MYH7, MYL2, and MYL3 in the soluble and filamentous fractions were quantified by mass spectrometry (MS). The abundances of soluble MYH6-MYH7, MYL2, and MYL3 increased (*P* < 0.001) with the concentration of KCl, as expected from the ionic strength-dependent disruption of the interactions between myosin tails (Figure 2A). More importantly, the abundance of the soluble MYH6-MYH7, MYL2, and MYL3 did not differ (*P* = 0.607) at any of the ionic conditions examined (Figure 2A). These data demonstrate that solubilization of the thick filaments in KCl solution in the presence of 10 μM mavacamten resulted in the release of whole myosin molecules rather than the selective release of the MYL2 from MHC.

### Mavacamten increased the abundance of soluble myosin extracted from intact muscle at room temperature (22°C).

To determine whether mavacamten increases the solubility of myosin molecules within the sarcomere, these data were compared with those generated from WT tissue triturated in the same solution in the absence of mavacamten. The abundances of soluble MYH6-MYH7, MYL2, and MYL3 increased (*P* < 0.001) with the concentration of KCl, but the abundance of each protein did not differ from one another (*P* = 0.961) at any of the ionic conditions examined (Figure 2B). These data were also indicative of the extraction of whole myosin molecules. However, comparison with the data collected with 10 μM mavacamten in the extraction solution demonstrated that mavacamten increased (*P* = 0.021) the solubility of myosin molecules across all KCl concentrations (Figure 2C). These data demonstrate that mavacamten enhances myosin solubility when extracted from ex vivo heart preparations.

### Mavacamten increased the solubility of myosin molecules, when polymerized at room temperature (22°C).

To confirm whether mavacamten increases myosin solubility, reductionist in vitro polymerization assays were performed. The thick filament proteins were purified from large ~30 mg pieces of WT mouse ventricular tissue (*n* = 3), and the molecules were completely solubilized using 600 mM KCl solution. A fraction of these samples was digested with trypsin, and the abundances of the proteins were determined by label-free quantitative MS and reported in Supplemental Table 1. In total, 44 proteins were identified by more than 2 peptides, and myosin molecules accounted for 78.9% ± 4.0% of the total protein abundance.

**Figure 3:**
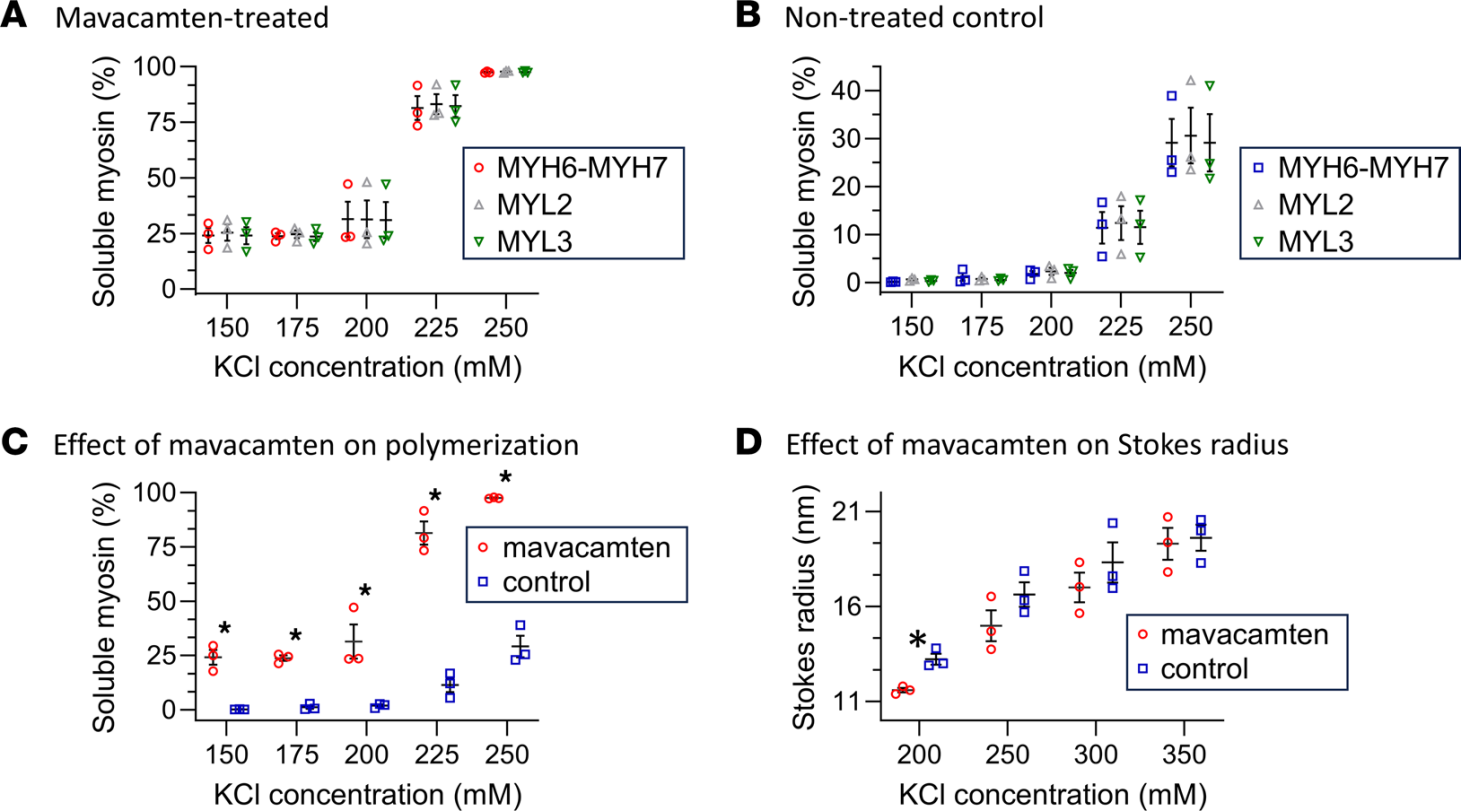
Quantification of the effect of mavacamten on myosin polymerization and folding at room temperature (22°C). (**A** and **B**) The average abundances (± SEM) of soluble MYH6-MYH7, MYL2, and MYL3 in KCl polymerization solution with (*n* = 3) (**A**) and without (**B**) 10 μM mavacamten (*n* = 3). (**C**) The average abundances (± SEM) of myosin in the KCl polymerization solution with and without 10 μM mavacamten. (**D**) The Stokes radii (± SEM) of MYL2-GFP within the soluble fraction of myosin in the presence and absence of 10 μM mavacamten (*n* = 3). Polymerization experiments were performed in the presence of 1 mM ATP (**C**), and MDS experiments were performed in the presence of 0.6 mM ATP (**D**). *P < 0.05 as determined by unpaired, 2-tailed *t* test.

Myosin filaments were repolymerized in the presence or absence of 10 μM mavacamten by diluting the protein at lower KCl concentrations (150–250 mM KCl) and incubating the samples at room temperature (22°C ± 2°C) for 2 hours. The soluble and filamentous proteins were separated by centrifugation at 18,000*g*, for 30 minutes at room temperature, and the abundances of MYH6-MYH7, MYL2, and MYL3 were quantified by MS. Both in the presence (Figure 3A) and absence (Figure 3B) of 10 μM mavacamten, the abundances of the MYH6-MYH7, MYL2, and MYL3 increased (*P* < 0.001) with KCl concentration. However, the abundances of the soluble MYH6-MYH7, MYL2, and MYL3 were the same at each KCl concentration (*P* = 0.9528 and *P* = 0.9301, respectively) within each group. Similar to that observed in the ex vivo extraction assays, the presence of mavacamten enhanced (*P* = 0.0165) the solubility of myosin when repolymerizing myosin into thick filaments (Figure 3C). These data further demonstrate that the presence of mavacamten shifts the equilibrium between filamentous and soluble myosin molecules toward the soluble pool.

### Mavacamten stabilizes the compact/folded conformation of myosin molecules, which are dissociated from thick filaments at room temperature (22°C).

We previously demonstrated that cardiac myosin molecules can adopt a compact/folded conformation when they are disassociated from the thick filaments at physiological ionic conditions using MDS (45). Therefore, we used the same approach to determine whether the presence of 10 μM mavacamten enhanced this compact/folded conformation. Thick filament proteins were purified from ~30 mg pieces of ventricular tissue (*n* = 3) from mice transduced with the MYL2-GFP AAV because MDS requires fluorescence detection. The molecules were completely solubilized in 400 mM KCl solution, and filament formation was induced in the presence or absence of 10 μM mavacamten by diluting the myosin to lower KCl concentrations (200–350 mM KCl) and incubating the samples at room temperature (22°C ± 2°C) for 2 hours. The Stokes radius of the MYL2-GFP in the soluble fraction, being indicative of the average size of the molecules, was determined by MDS at room temperature (22°C ± 2°C). The smallest radius (11.7 ± 0.1 nm) was observed at the lowest KCl concentration, and the largest radius (19.6 ± 0.8 nm) was observed at the highest KCl concentration (Figure 3D). These radii were much larger than the 3.1 nm radius predicted for MYL2-GFP (46), further suggesting that MYL2 remains strongly bound to MHC when disassociated from the thick filaments. The smallest radius was similar to the 12.5 nm radius determined for smooth muscle myosin molecules in a compact/folded (10 S) conformation, while the largest was similar to the 18.5 nm radius when in the extended (6 S) conformation (47, 48). The intermediate radii were indicative of mixtures of these conformations (45). Most importantly, the radii determined in the presence of 10 μM mavacamten across these KCl concentrations were smaller (*P* = 0.012) than those observed in the absence of mavacamten as determined by 2-tailed, paired *t* test (Figure 3D), noting that only the radii at the lowest KCl concentrations (200 mM KCl) were statistically (P< 0.01) different in the presence of 10 μM mavacamten when compared using a 2-tailed, unpaired *t* test (Figure 3D).

### The folding of myosin molecules into a compact conformation regulates myosin solubility.

**Figure 4:**
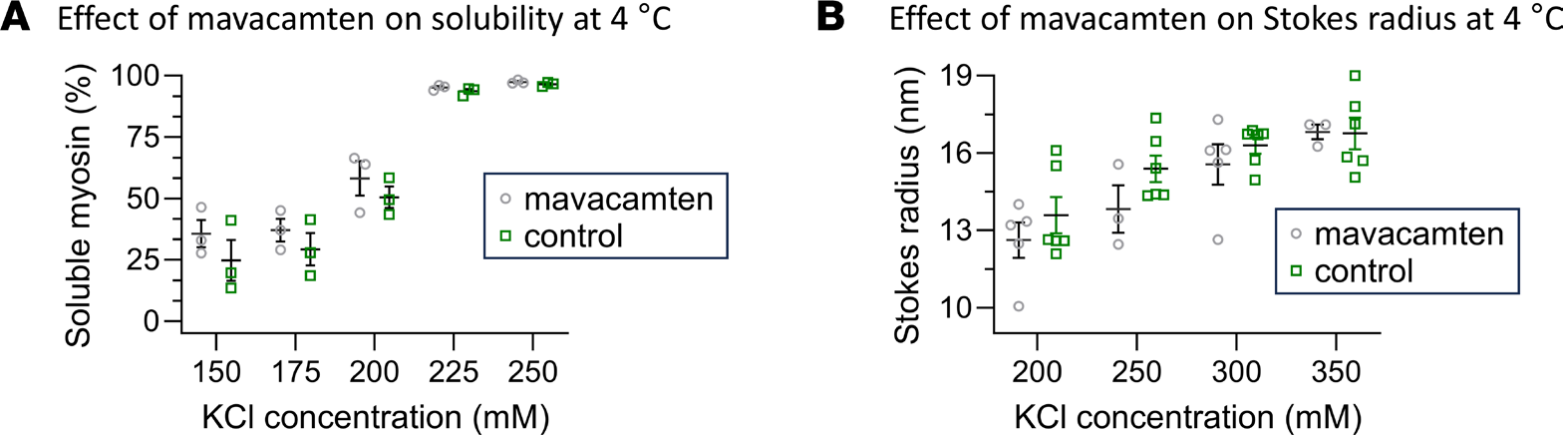
Quantification of the effect of temperature and mavacamten on myosin polymerization and folding. (**A**) The average abundances (± SEM) of soluble myosin in KCl polymerization solution in the presence and absence of 10 μM mavacamten at 4°C (*n* = 3). (**B**) The Stokes radius (± SEM) of myosin molecules in presence of 10 μM mavacamten at 4°C (*n* = 3). Polymerization experiments were performed in the presence of 1 mM ATP, and MDS experiments were performed in the presence of 0.6 mM ATP.

The FRAP, extraction, polymerization, and MDS assays were all carried out at room temperature (22°C ± 2°C). Both myosin dissociation (49) and function of mavacamten (27) have been demonstrated to be affected by temperature. Therefore, we took advantage of these temperature dependencies to further explore the relationships between myosin solubility and folding. First, the myosin polymerization assays were repeated at 4°C. The presence of 10 μM mavacamten had no apparent effect on myosin solubility (*P* = 0.8919) at 4°C when compared with solubility in the absence of mavacamten at the same temperature (Figure 4A). Next, the MDS assays were repeated at 4°C. The presence of 10 μM mavacamten had no apparent effect on the Stokes radii at 4°C when compared with those in its absence of mavacamten at the same temperature (Figure 4B). These data demonstrate that the effect of mavacamten on myosin solubility and folding is temperature dependent and that the degree of myosin solubility is correlated with the compact/folded conformation of myosin molecules when they are disassociated from the thick filaments.

### Mavacamten slows the reentry of myosin molecules into the thick filament.

**Figure 5:**
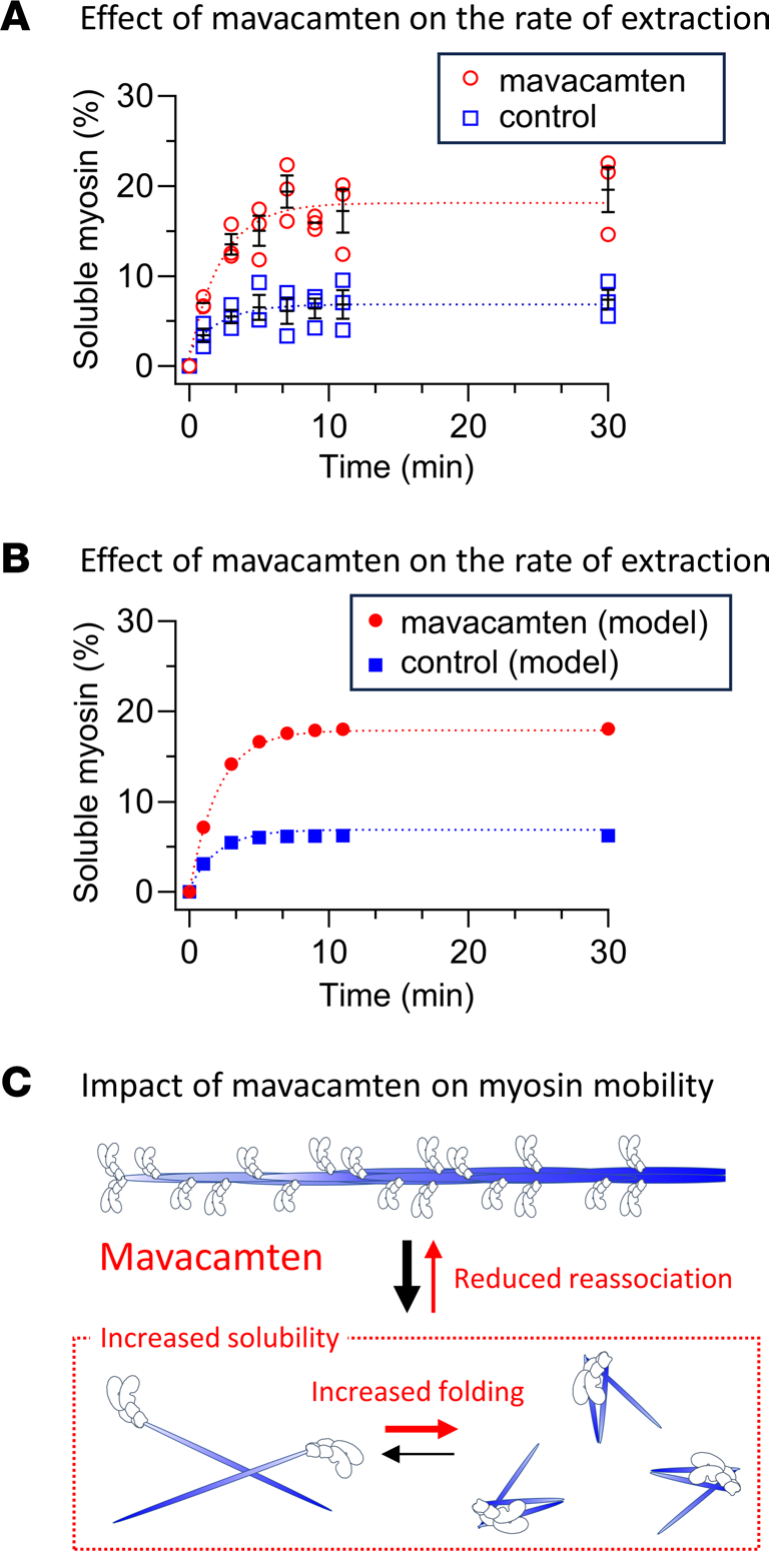
Quantification of the effect of mavacamten on the rate of extraction of myosin molecules from intact cardiac muscle at room temperature (22°C). (**A**) The average abundances (± SEM) of soluble myosin molecules extracted from intact muscle using 225 mM KCl extraction buffer with (*n* = 3) and without 10 μM mavacamten (*n* = 3) versus time. All experiments were performed in the presence of 0.7 mM ATP. Data were fitted with a single exponential; dashed lines. The plateaus differed (*P* = 0.0018) but the rate constants (k) did not (*P* = 0.5235), as determined by extra sum-of-squares F tests. (**B**) The theoretical abundance of myosin was determined to be extracted from the tissue using an analytical model that tuned the rate of reentry of myosin molecules into the filament. Solid symbols are the theoretical abundances. Dashed lines are the fits from **A**. (**C**) Schematic demonstrating the effect of mavacamten on myosin solubility by affecting folding and reentry.

The effect of mavacamten on myosin mobility, solubility, and folding may contribute to an increase in the rate of release of myosin molecules from the thick filament and/or a reduction in their rate of reassociation. To distinguish between these mechanisms, we performed timed extraction assays. Small, ~1 mg pieces of ventricular tissue were triturated for 30 seconds at room temperature (22°C ± 2°C) in 225 mM KCl solution containing 0.1% Triton X-100 detergent with and without 10 μM mavacamten. The soluble proteins were removed 1, 3, 5, 7, 9, 11, or 30 minutes after the sample was placed in the solution, and the abundance of myosin in each fraction was quantified by MS. The change in the abundance of soluble myosin extracted in the presence and absence of 10 μM mavacamten was fitted with a single exponential (Figure 5A). The rate constants being 0.428 (±0.215–0.837) min^–1^ in the presence and 0.6299 (±0.367–1.056) min^–1^ in the absence of 10 µM mavacamten, were similar (*P* = 0.5235), while the plateaus being 18.2% (±16.0%–21.2%) and 6.7% (±6.2%–7.3%) differed (*P* = 0.0018).

To interpret these data, we generated an analytical model based on the fundamental relationship between equilibrium (K_eq_) and rate constants, such that the mole fraction of soluble/filamentous myosin was equal to the rate constant of disassociation (k_out_) divided by the rate constant of reassociation (k_in_) (Figure 5B). The k_out_ and mole fraction of soluble to filamentous myosin were used to estimate k_in_ in the presence and absence of 10 μM mavacamten (Figure 5B). This resulted in the k_in_ in the presence of mavacamten being > 4-fold less than in the absence of mavacamten. Using these rate constants, the analytical model could recapitulate the data (Figure 5B).

## Discussion

Striated muscle contractility is primarily regulated by the calcium-dependent access of myosin to the thin filament, but emerging evidence demonstrates that the force and velocity of each contraction is modulated by the availability of myosin heads protruding from the thick filament backbone (9, 10, 50, 51). An excess in the availability of myosin heads is proposed to be pathologic, resulting in HCM. Mavacamten is an effective small molecule for the treatment of HCM, which reduces cellular force generation and ventricular hypercontractility (16, 30, 31, 40). Experimental evidence from in vitro assays demonstrates that mavacamten shifts myosin heads into a SRX biochemical state (14, 15, 26, 27) and/or stabilizes the intra- and intermolecular interactions between myosin molecules within the thick filament (15, 41). These biochemical and structural effects reduce the ability of myosin to bind actin. However, data from our lab (44, 45) and others (52–59) demonstrate that the structure of the thick filament is highly dynamic in its native environment. One interpretation of these data is that myosin molecules disassociate from the thick filament, undergo diffusive mobility, and reincorporate into distal locations in the same or neighboring thick filaments within the sarcomere on the minute timescale (45). Based on the current working knowledge of mavacamten, we aimed to further test this interpretation. We hypothesized that the stabilization of intra- and intermolecular contacts within thick filaments would slow the dissociation of myosin from the filaments and reduce its apparent mobility within the sarcomere.

To test the hypothesis, we utilized AAV transduction to replace a fraction of the endogenous MYL2 in adult mouse hearts with MYL2 having GFP fused to its C-terminus (MYL2-GFP). We previously demonstrated that the MYL2-GFP was properly localized to myosin heads within the thick filament had no detectable affect on cardiac mass or the ability of myosin to translocate actin filaments in vitro (45). However, the MYL2-GFP appeared highly mobile within the confines of sarcomeres, when quantifying FRAP after MYL2-GFP was photobleached within portions of sarcomeres in intact heart preparations (45).

In the current report, we used this same approach to determine whether incubation of intact heart preparations in 10 μM mavacamten affected myosin mobility. The preparation was immediately submerged in Tyrode’s solution containing 10 μM mavacamten for 1 hour to ensure permeability of the small molecule (60). Unexpectedly, incubation in mavacamten prior to imaging resulted in a > 5-fold increase in the rate of FRAP, as compared with that observed in the absence of mavacamten (Figure 1E). This result was surprising. While structural studies demonstrated that mavacamten folds fragments of soluble myosin molecules, these studies assumed this folding only occurs while myosin was located within the thick filament (15, 26). Therefore, mavacamten has been used in thick filament (4) and whole muscle preparations (5) to presumably limit molecular disorder to enhance the resolution of electron micrographic reconstructions.

For the MYL2-GFP to be mobile, molecules must be released from the thick filament, must diffuse within the space between filaments, and must be reincorporated into a distal location of the same or a neighboring thick filament within the sarcomere. One possible conclusion from our previous report quantifying MYL2-GFP mobility in the absence of mavacamten was that MYL2-GFP mobility resulted from the mobility of whole myosin molecules (45). However, we could not eliminate the possibility that the MYL2-GFP was exchanging off and on MHC that remained strongly bound within the thick filaments (45). To test whether mavacamten enhanced the exchange of the MYL2-GFP or whole myosin molecules between filaments, we determined whether incubation of intact muscle (Figure 2) or purified thick filament proteins (Figure 3) in 10 μM mavacamten resulted in the preferential solubility of MYL2. The relative abundances of MYH6-MYH7, MYL2, and MYL3, the ventricular alkali isoform, were the same in the soluble and filamentous fractions in the presence and absence of mavacamten, when myosin was extracted from muscle (Figure 2, A and B) or repolymerized into thick filaments in vitro (Figure 3, A and B) over the entire range of ionic conditions. These data provide evidence that the MYL2 remains strongly bound to the heavy chain when myosin molecules are disassociated from the thick filaments in the presence of mavacamten. These data are consistent with prior structural studies that demonstrate that mavacamten folds the myosin heads onto the proximal (S2) portion of the coiled-coil tail of myosin fragments (15, 26), rather than removing the MYL2 from the heavy chain. However, it is still possible that the MYL2 may exchange between MHCs on a timescale that is not observed in our assays.

The solubility of myosin molecules was enhanced in the presence of 10 μM mavacamten both when the intact ventricular tissue was demembranated with Triton X-100 and exposed to ionic solutions to disassociate molecules from the thick filaments (Figure 2C) and when soluble myosin molecules were repolymerized in vitro by reducing the ionic conditions (Figure 3C). The difference in the absolute magnitude of myosin solubility observed in these 2 assays may be explained by the difference in approaches. In the extraction assay, additional structural proteins such as myosin-binding protein C, titin, and myomesin are incorporated into the native thick filaments, which may alter the extraction of myosin; alternatively, not all cells may have been fully demembranated. In support of the latter point, the absolute abundance of myosin extracted in the assay was less than that previously reported using a more aggressive extraction approach that involved extended trituration, rocking, and centrifugation to separate the fractions (44). Regardless of the difference in magnitude, both assays demonstrate that mavacamten shifts the equilibrium between myosin existing in thick filaments toward a pool of soluble myosin molecules.

Our previous study demonstrated that soluble myosin molecules adopt a compact/folded conformation when disassociated from the thick filaments at physiological ionic conditions (45). To test whether mavacamten affects this folding, we quantified the Stokes radius of soluble MYL2-GFP containing myosin molecules in the presence and absence of mavacamten (Figure 3D). The smallest Stokes radius determined by MDS, being 11.7 ± 0.1 nm, was considerably larger than the 3.1 nm radius predicted from free MYL2-GFP (45, 46) and consistent with the size of a compact/folded myosin molecule (45). The largest radius, being 19.6 ± 0.8 nm, was consistent with a fully extended myosin molecule, and the intermediate radii were indicative of mixtures of conformations (45). The radii measured in the presence of 10 μM mavacamten were smaller across the entire range of ionic conditions, with the greatest difference at the lowest ionic conditions (Figure 3D). Our data demonstrate that mavacamten may play a physiological role in stabilizing the compact/folded structure of myosin molecules that are disassociated from the thick filaments. This shift in the equilibrium would reduce the average number of myosin molecules in the thick filaments, necessary for the generation of contractile force.

Myosin solubility (49), thick filament structure (61), and the function of mavacamten (27) have been demonstrated to be affected by temperature. Our initial experiments were performed at room temperature (22°C ± 2°C), so we repeated several assays at 4°C to further explore the relationships between myosin solubility and folding. Mavacamten had no effect of solubility (Figure 4A) or folding (Figure 4B) at 4°C, further demonstrating a mechanistic link between myosin solubility and the folding of myosin monomers. This relationship between solubility and folding was similar to that demonstrated for both smooth (47, 48, 62) and nonmuscle myosin (48, 63). Most importantly, mavacamten affected both myosin solubility and folding at closer to physiologically relevant temperatures, with the latter capable of enhancing the rate of myosin diffusion in the FRAP assays (Figure 1E).

The enhanced rate of myosin mobility determined by FRAP is dependent on the rate of myosin diffusion as well and the rates of release of myosin molecules from the thick filament and their reassociation into a distal location within the same or a neighboring filament within the sarcomere. To determine whether mavacamten enhanced solubility by increasing the rate of release of myosin from the thick filament and/or by decreasing the rate of reentry into a thick filament, soluble myosin was extracted from demembranated cardiac tissue, but the time allowed for diffusion of molecules out of the tissue was limited (Figure 5A). Both in the presence and absence of mavacamten, the data were well described by single exponentials (Figure 5A). The plateau of the fit was 2.7-fold greater in the presence of mavacamtem (Figure 5A), as expected from the longer extraction assays (Figure 2C). However, the rate constants of the fits were similar in the presence and absence of mavacamten, suggesting that the rate of release was not affected. Therefore, the primary effect of mavacamten on myosin solubility appears to result from a decrease in the rate of reassociation of myosin with the thick filaments. Such a decrease in the rate of reassociation is at odds with the fact that there would be more available binding sites within the thick filament due to the shift in the equilibrium away from the filaments. This suggests that the folding of the myosin monomers into the compact configuration likely blocks intermolecular interactions between tails of myosin molecules, which are essential for filament polymerization and stability.

Taken together, our data collectively demonstrate that a fraction of myosin molecules are continually released from the thick filaments, diffuse in the free space in a compact/folded conformation, before reentering the same, or a neighboring thick filament in a distal location of the sarcomere in intact muscle. Although mavacamten may affect the folding of myosin heads onto the thick filament backbone, this does not appear to affect the rate of disassociation of the molecules from the thick filament within the resolution of our assays. The primary effect of mavacamten appears to be the stabilization of myosin molecules in their compact/folded conformation once they are dissociated from the thick filaments, and this ultimately shifts the equilibrium between the giant filamentous macromolecular complexes and soluble monomers (Figure 5C). This mechanism is different than that observed for high ionic conditions and pH, as well as low temperatures, which all appear to shift the equilibrium by altering the rate of disassociation of myosin molecules from the thick filaments (49).

While the compaction/folding of the soluble myosin molecules would enhance the rate of diffusion within the space between the filaments by limiting the Stokes radius, the > 5-fold increase in the rate of myosin mobility in the presence of mavacamten is likely driven by an inability of myosin to rapidly reassociate with the thick filament. This would result in a net reduction in the average number of myosin molecules localized within the thick filaments at any moment in time. The hypothesis that the backbone of the thick filament is loose, which can allow for the modulation of myosin number, is supported by recent elegant cryogenic electron micrographs of thick filaments and intact sarcomeres that demonstrate gaps in the backbone of the thick filaments in the presence of mavacamten (4, 5).

While the current models suggest that mavacamten elicits its functional effects by folding the myosin heads back onto the thick filament to limit the rate of ATP hydrolysis, the vast majority of the heads in electron micrographs of relaxed cardiac muscle thick filaments have been demonstrated to be folded on to the thick filament backbone even in the absence of mavacamten (23, 25). Our new data suggest that it is entirely possible that the effect of mavacamten on muscle contractility is multifaceted and extends beyond the function of the myosin head within the thick filament. The shift in the equilibrium away from filaments, and toward the pool of soluble monomers, would effectively have a similar effect on the number of functional myosin heads available to bind to the actin filament in vivo. This in turn limits the ensemble behavior that is necessary for the function of cardiac muscle myosin, thus reducing ATPase activity and force transduction, as previously observed in functional studies using in vitro molecular assays (39, 64), cardiac muscle cells (65, 66), and intact tissue (39, 40). Thus, these new data regarding the effect of mavacamten on myosin mobility and solubility should be considered in future interpretations of biochemical and functional data, as the soluble myosin may contribute to the super relaxed state of myosin. Such an interpretation may alter our understanding of how mavacamten limits cardiac contractility in the treatment of HCM.

## Methods

### Sex as a biological variable.

Our study examined male and female FVB WT mice purchased from The Jackson Laboratory, and sex was not considered as a biological variable.

### AAV design, production, and use.

Mouse *MYL2* was fused in frame with pENN.AAV. *TNNT2*.PI.eGFP.WPRE.rBG to generate the recombinant chimera AAV9/pENN.AAV.*TNNT2*.*MYL2*.eGFP.WPRE.rBG as previous described (44, 45). This was cotransfected with pAAV2/9.45 (serotype 2 rep coding sequences, serotype 9.45 cap coding sequences) and pHelper plasmids (Stratagene) into AAV-293 cells utilizing PEI to generate the AAV as previously described (44, 45). AAV titers were 1 × 10^13^ vp/mL.

### Transfection of mice with AAV and euthanasia.

Expression of myosin regulatory light chain 2, cardiac ventricular isoform, with GFP fused to its C-terminus (MYL2-GFP) was induced by the transduction of 3- to 6-month-old WT mice via retro-orbital injection with 100 μL of 1 × 10^13^ vp/mL AAV. To allow for robust MYL2-GFP expression, mice were anesthetized with isoflurane and euthanized by cervical dislocation and exsanguination, 12 ± 2 days after transduction. Whole hearts were harvested for 2-photon imaging and/or protein purification.

### Ex vivo heart preparation for 2-photon imaging.

Immediately after the heart was removed, the left ventricle was splayed open with scissors, and the whole heart was pinned to a Sylgard-coated (Dow Corning) dish to expose the papillary muscle. The preparation was covered in 10 mL of Tyrode’s solution (137 mM NaCl, 2.7 mM KCl, 1 mM MgCl_2_, 0.2 mM Na_2_HPO_4_, 12 mM NaHCO_3_, 5.5 mM glucose, 10 μM ATP, ~1 μM creatine phosphokinase, pH 7.4), with or without 10 μM mavacamten (Cayman Chemicals). The hearts were incubated in the solution for 1 hour at room temperature (22°C ± 2°C) prior to imaging and remained submerged in the solution for the duration of the imaging experiments (*n* = 8). All chemicals were from Sigma-Aldrich, unless noted.

### Two-photon imaging and photobleaching.

Fluorescence imaging was performed using a laser-scanning Zeiss LSM-7 multiphoton system with a 20× Plan Apo 1.0 NA DIC VIS-IR water immersion lens as previously described (45). To quantify FRAP within single sarcomeres, the MYL2-GFP fluorescence was quenched in small randomly selected volumes of the images, being ~1–2 μm in depth, using high-intensity excitation. FRAP was detected by capturing an image every 30 seconds for up to 30 minutes. More images were collected in each image series for the control hearts, which were not treated with mavacamten, because the FRAP was noticeably slower while recording the images. *Z* stacks were collected in 1 μm steps after imaging to ensure recovery was not an artifact of drift in the *z* axis of the focal plane.

### Two-photon image analyses.

Images were viewed and intensities of pixels of interest (102.5 nm/pixel) were quantified using Fiji (ImageJ; NIH) (67). To identify the longitudinal (Z discs) and axial (myofibril width) boundaries of single sarcomeres, fluorescence intensity distribution profiles were generated along the longitudinal and transverse axis striations present in the images prior to photobleaching. This approach has been described in detail for FVB WT hearts transduced with the MYL2-GFP AAV (45). To quantify FRAP, fluorescence intensity distribution profiles were generated from small ROIs that contained a sarcomere in which only a fraction of the MYL2-GFP molecules were photobleached, and a nonphotobleached sarcomere as previously described (45). Fluorescence intensities were normalized to unbleached MYL2-GFP in each image to account for normal photobleaching, which occurred while imaging. The normalized intensity for each frame within each ROI was determined using Equation 1

 (Equation 1)
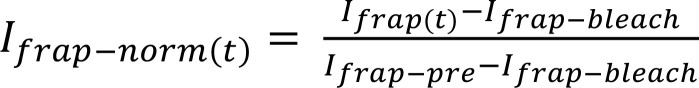


In which *I_frap-norm(t)_* is the normalized intensity of the ROI at time *t*, *I_frap_* is the raw intensity of the ROI, *I_frap-bleach_* is the raw intensity of the ROI immediately after photobleaching, and *I_frap-pre_* is the raw intensity of the ROI prior to photobleaching. The average intensities within each ROI were plotted with respect to time. The average intensity ± SEM for each condition was fitted with a single exponential regression using Prism 10. The rate constants, half-lives, and plateaus of recovery (±95% CIs) and coefficient of determination (*R*^2^) were reported for each fit. Statistical differences between the control and mavacamten-treated groups were determined using extra sum-of-squares F test, and the *P* value was reported.

### Myosin extraction assays.

To determine whether mavacamten affects the solubility of myosin molecules in intact tissue, small 1–2 mg pieces of mouse left ventricular tissue were demembranated with Triton X-100, soluble proteins were extracted in extraction solution containing KCl, and the abundance of protein was quantified using liquid chromatography–MS (LC-MS). Under all conditions, the tissue was mechanically triturated for 1 minute with forceps in a dissection chamber with 400 μL of extraction solution. The solution contained: KCl, 1 mM EGTA; 25 mM imidazole, 4 mM MgCl_2_ [pH 7.4], 0.4 mg/mL creatine phosphate, 0.7 mM ATP, 7 mM DTT, 0.1% Triton X-100 detergent, and 5 μg/mL bovine serum albumin (BSA) with or without 10 μM mavacamten; the KCl concentration varied (150, 175, 200, 225, and 250 mM) as described. To probe steady-state extraction conditions at each KCl concentration, the samples were allowed to sit at room temperature (22°C ± 2°C) for 30 minutes, and then the solution was transferred to a 1.5 mL microcentrifuge tube using a gel-loading pipette tip. To probe the effect of time on extraction in 225 mM KCl extraction solution, each sample was allowed to sit for 1, 3, 5, 7, 9, 11, and 30 minutes after trituration, and then the solution was transferred to a 1.5 mL microcentrifuge tube using a gel-loading pipette tip. In all experiments, the pellets were resuspended in the same volume of extraction solution and transferred to a 1.5 mL microcentrifuge tube. The BSA in the extraction solution served as an internal standard for quantification by LC-MS.

To remove the Triton X-100 in preparation for LC-MS, the samples were dried down by centrifugal evaporation and then resuspended in 500 μL of 5% trichloroacetic acid (TCA) to precipitate proteins. Precipitated proteins were pelleted and collected by centrifugation at 18,800*g* for 5 minutes. The supernatants were discarded. Each sample was resuspended and precipitated in 500 μL of 5% TCA twice more to remove residual detergent. For protein quantification by LC-MS, each sample was resuspended in 75 μL of 50 mM ammonium bicarbonate with the addition of 0.75 μL of 1M DTT and heated for 10 minutes at 100°C to reduce disulfide bonds. The samples were alkylated by addition of 22.4 μL of 0.1M iodoacetamide and incubation for 30 minutes in the dark (room temperature [RT]). Each sample was digested for 18 hours at 37°C by addition of 25 μL of a 0.2 μg/μL trypsin (Promega) in 50 mM ammonium bicarbonate solution. Following digestion, 100 μL of 7% formic acid in 50 mM ammonium bicarbonate was added to inactivate trypsin, and samples were dried down by centrifugal evaporation. The samples were resuspended in 0.1% TFA, centrifuged for 5 minutes at 18,800*g*, and the supernatant was analyzed by LC-MS.

For each experimental condition, the extraction assays were performed in triplicate. The average ± SEM was graphed using GraphPad Prism 10. The steady-state extraction data were plotted as the percent soluble myosin at each concentration of KCl. Differences in the abundances of MYH6-MYH7, MYL2, and MYL3, the ventricular alkali isoform, were tested using a 2-way ANOVA. Significant differences between data sets and at individual KCl concentrations were determined using 2-tailed, paired and unpaired *t* tests. The timed extraction data were fitted with a single exponential, and significant differences between rate constants and plateaus were determined using an extra sum-of-squares F test and, the *P* value was reported.

### Myosin filament polymerization assays.

To determine whether mavacamten affects the polymerization of myosin molecules, thick filament proteins were isolated from ~30 mg pieces of ventricular tissue from the apex of FVB WT hearts (*n* = 3); filaments were formed under a range of KCl concentrations. The abundances of all proteins isolated from the tissue, and the abundances of soluble and filamentous myosin in each experimental sample, were quantified by LC-MS, as previously described (44, 68).

Briefly, to isolate the thick filament proteins, the apex of the heart was removed and homogenized in homogenization buffer (300 mM KCl, 150 mM K_2_HPO_4_, 10 mM Na_4_P_2_O_7_, 1 mM MgCl_2_, 2 mM DTT, 1 mM ATP [pH 6.8]) using a glass homogenizer. Cell debris was pelleted at 150,000*g* for 1 hour at 4°C, and the supernatant was decanted. The supernatant was then diluted 5,000× in 2 mM DTT and incubated at 4°C for ≥ 1 hour for myosin filament formation. Myosin filaments were pelleted at 50,200*g* at 4°C for 20 minutes and solubilized in high KCl buffer (600 mM KCl, 25 mM imidazole, 1 mM EGTA, 4 mM MgCl_2_, 6.7 mM DDT, 0.7 mM ATP, 4 μg/mL creatine phosphate) overnight. Purified myosin (~2 mg/mg) was then diluted to ~0.4 mg/mL in a mixture of high KCl buffer and low KCl buffer (25 mM KCl, 25 mM imidazole, 1 mM EGTA, 4 mM MgCl_2_, 6.7 mM DDT, 0.7 mM ATP, 4 μg/mL creatine phosphate) to achieve desired concentrations of KCl. Where applicable, 10 μM mavacamten (Cayman Chemical Company) was added to the sample, and each sample was incubated overnight at 4°C.

The following day, filaments were separated from soluble myosin by centrifugation, and the supernatant was decanted into a new 1.5 mL microcentrifuge tube. For experiments performed at RT, the samples were incubated at 22°C ± 2°C for 2 hours prior to centrifugation at 18,000*g* for 30 minutes, at 22°C. For experiments performed at 4°C, the samples were immediately centrifuged at 18,000*g* for 30 minutes, at 4°C, noting that the pH of the solution was 7.39 at 22°C and 7.53 at 4°C. A 10 μL aliquot of 0.1M BSA was added to supernatant and pelleted fractions for normalization. The samples were then dried down by centrifugal evaporation in an Eppendorf Vacufuge.

For the identification and quantification of proteins isolated from the tissue, and in the filamentous and soluble fractions, samples were resuspended in 75 μL of 50 mM ammonium bicarbonate with the addition of 5 μL of 0.1M DTT and heated for 10 minutes at 100°C. A 10.4 μL aliquot of 0.1M iodoacetamide was added to for 30 minutes in the dark (RT). A 25 μL aliquot of a 0.2 μg/μL trypsin (Promega) in 50 mM ammonium bicarbonate solution was added to each sample, and they were digested for 18 hours at 37°C. Following digestion, a 100 μL aliquot of 7% formic acid in 50 mM ammonium bicarbonate was added to inactivate trypsin, and samples were dried down by centrifugal evaporation. The samples were resuspended in 0.1% trifluoroacetic acid (TFA), centrifuged for 5 minutes at 18,800*g*, and the supernatant was analyzed by LC-MS.

For each experimental condition, the polymerization assay was performed in triplicate from 3 independent protein purifications. The average ± SEM was graphed using GraphPad Prism 10. Significant differences in the abundances of MYH6-MYH7, MYL2, and MYL3, the ventricular alkali isoform, were tested using a 2-way ANOVA. Significant differences between data sets and individual KCl concentrations were determined using a 2-tailed paired or unpaired *t* test and the *P* values were reported

### Quantification of protein abundances by LC-MS.

Peptides were separated by injection of 20 μL of each sample onto an Acquity UPLC HSS T3 column (100 Å, 1.8 μm, 2.1 mm × 150 mm) (Waters Corporation) attached to an UltiMate 3000 ultra–high pressure LC (UHPLC) system (Dionex). The UHPLC effluent was directly infused into a Q Exactive Hybrid Quadrupole-Orbitrap mass spectrometer through an electrospray ionization source (Thermo Fisher Scientific). Data were collected in data-dependent MS/MS mode, with the top 5 most abundant ions being selected for fragmentation, as previously described (44, 68).

To quantify the abundance of proteins in each sample, the .raw files were examined with an automated routine using Thermo Proteome Discoverer 2.2.0.388 (PD 2.2) as described (44, 68). The MS data were searched using Sequest HT against a Mus musculus database (containing 74,085 sequences; downloaded 02/09/15 from Uniprot) and bovine albumin (Uniprot accession P02769; downloaded 10/12/18). The search parameters allowed for several dynamic modifications including: carbamidomethyl (Cys), oxidation (Met, Pro), dioxidation (Met), and phosphorylation (Ser, Thr, Tyr). The average abundance of the top 3 ionizing peptides resulting from the digestion of each protein of interest was divided by the average abundance of the top 3 ionizing peptides resulting from the digestion of all proteins in the purified protein prep or BSA in the samples from the solubility assays. The percentage of each protein of interest in the soluble fraction was defined as the relative abundance of the protein in the soluble fraction divided by the summed abundance of the protein in both fractions.

### MDS.

To determine whether mavacamten affects the folding of myosin molecules into a compact conformation, the Stokes radius of soluble myosin molecules was determined by MDS. This technique relies on fluorescence detection, so myosin molecules were isolated from ~30 mg pieces of ventricular tissue from the apexes of 3 AAV-transduced hearts, as described for the filament polymerization assays. However, following pelleting at 25,000 rpm (4°C, 20 minutes), myosin was solubilized in a high KCl storage buffer (400 mM KCl, 10 mM Tris, 1 mM DTT, 0.6 mM ATP [pH 7.4]). Soluble myosin was then diluted in low KCl tris buffer (10 mM Tris, 1 mM DTT, 0.6 mM ATP [pH 7.4]) to achieve desired concentrations of KCl for polymerization (200, 250, 300, and 350 mM) and then incubated overnight in the presence or absence of 10 μM mavacamten at 4°C.

The following day, the samples were either incubated at 22°C ± 2°C for 2 hours or immediately used, noting that the pH of the solution was 7.42 at 22°C and 7.67 at 4°C. The samples were centrifuged at 18,000*g* for 30 minutes at either 22°C or 4°C. The supernatants were retained at their respective temperature for use in the MDS assay, and filament-containing pellets were discarded. A 3.5–4 μL aliquot of soluble myosin was loaded on a microfluidic chip (Fluidic Analytics) for MDS analysis. Despite the difference in starting temperatures, the MDS measurements could only be performed at room temperature on a Fluidity One-M device (Fluidic Analytics). The instrument used a 488 nm LED for excitation, the size range was set at 2–20 nm, and the viscosity setting was 1, as previously described (45). The MDS measurements were completed within 10 seconds of loading the chip. The Stokes radii (± SEM) were plotted using GraphPad Prism 10 and fitted with a 1-phase association, single exponential curve. Statistical differences between data sets were determined using a 2-tailed, paired *t* test, and the *P* value was reported. Statistical differences at individual KCl concentrations were also measured using a 2-tailed, unpaired *t* test, and the P values were reported.

### Analytical modeling of filament depolymerization/polymerization kinetics.

The depolymerization rate constants and plateaus obtained from fitting the timed extraction assay were used to determine the polymerization rate constant. Using the fundamental relationship between rate constants and concentration at equilibrium, the simplified equation was used:

 (Equation 2)
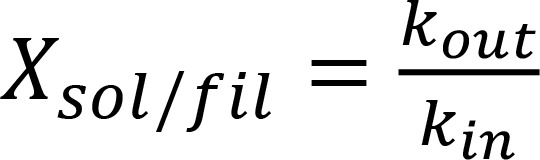


in which *X_sol/fil_* is the experimentally obtained mole fraction of soluble to filamentous myosin at equilibrium, *k_out_* is the experimentally obtained rate constant of disassociation of myosin molecules from the thick filament, and *k_in_* is the predicted rate constant for reassociation of myosin with the thick filament at equilibrium.

### Statistics.

The significance of differences in measurements were determined using statistical analyses described in each subsection, and *P* values are reported in the Results and figure legends.

### Study approval.

Mice were handled, cared for and euthanized in accordance with protocols approved by the University of Vermont IACUC.

### Data availability.

Values for all data points in graphs are reported in the Supporting Data Values file and are available from the corresponding author upon request.

## Author contributions

CMK designed research studies, conducted experiments, acquired data, analyzed data, and wrote the manuscript. JLM designed and produced reagents and edited the manuscript. MJP designed research studies, analyzed data, and edited the manuscript.

## Supplementary Material

Supplemental data

Supplemental video 1

Supporting data values

## Version 1 03/14/2024

In-Press Preview

## Version 2 04/22/2024

Electronic publication

## References

[1] Huxley HE (1963). Electron microscope studies on the structure of natural and synthetic protein filaments from striated muscle. J Mol Biol.

[2] Lowey S (1967). Proteolytic degradation of myosin and the meromyosins by a water-insoluble polyanionic derivative of trypsin: properties of a helical subunit isolated from heavy meromyosin. J Mol Biol.

[3] Vikstrom KL (1997). The vertebrate myosin heavy chain: genetics and assembly properties. Cell Struct Funct.

[4] Dutta D (2023). Cryo-EM structure of the human cardiac myosin filament. Nature.

[5] Tamborrini D (2023). Structure of the native myosin filament in the relaxed cardiac sarcomere. Nature.

[7] Holmes KC (1997). The swinging lever-arm hypothesis of muscle contraction. Curr Biol.

[8] Tang W (2021). Cardiomyopathy mutations impact the actin-activated power stroke of human cardiac myosin. Biophys J.

[9] Brunello E (2023). Activation of skeletal muscle is controlled by a dual-filament mechano-sensing mechanism. Proc Natl Acad Sci U S A.

[10] Brunello E, Fusi L (2024). Regulating striated muscle contraction: through thick and thin. Annu Rev Physiol.

[11] Stewart MA (2010). Myosin ATP turnover rate is a mechanism involved in thermogenesis in resting skeletal muscle fibers. Proc Natl Acad Sci U S A.

[12] Naber N (2011). Slow myosin ATP turnover in the super-relaxed state in tarantula muscle. J Mol Biol.

[13] Hooijman P (2011). A new state of cardiac myosin with very slow ATP turnover: a potential cardioprotective mechanism in the heart. Biophys J.

[14] McNamara JW (2015). The role of super-relaxed myosin in skeletal and cardiac muscle. Biophys Rev.

[15] Anderson RL (2018). Deciphering the super relaxed state of human β-cardiac myosin and the mode of action of mavacamten from myosin molecules to muscle fibers. Proc Natl Acad Sci U S A.

[16] Nag S, Trivedi DV (2021). To lie or not to lie: super-relaxing with myosins. Elife.

[17] Alamo L (2017). Lessons from a tarantula: new insights into muscle thick filament and myosin interacting-heads motif structure and function. Biophys Rev.

[18] Jung HS (2008). Head-head and head-tail interaction: a general mechanism for switching off myosin II activity in cells. Mol Biol Cell.

[19] Burgess SA (2007). Structures of smooth muscle myosin and heavy meromyosin in the folded, shutdown state. J Mol Biol.

[20] Jung HS (2011). Role of the tail in the regulated state of myosin 2. J Mol Biol.

[21] Wendt T (2001). Three-dimensional image reconstruction of dephosphorylated smooth muscle heavy meromyosin reveals asymmetry in the interaction between myosin heads and placement of subfragment 2. Proc Natl Acad Sci U S A.

[22] Woodhead JL (2005). Atomic model of a myosin filament in the relaxed state. Nature.

[23] Zoghbi ME (2008). Three-dimensional structure of vertebrate cardiac muscle myosin filaments. Proc Natl Acad Sci U S A.

[24] Alamo L (2008). Three-dimensional reconstruction of tarantula myosin filaments suggests how phosphorylation may regulate myosin activity. J Mol Biol.

[25] Al-Khayat HA (2013). Atomic model of the human cardiac muscle myosin filament. Proc Natl Acad Sci U S A.

[26] Chu S (2021). Direct detection of the myosin super-relaxed state and interacting-heads motif in solution. J Biol Chem.

[27] Walklate J (2022). Exploring the super-relaxed state of myosin in myofibrils from fast-twitch, slow-twitch, and cardiac muscle. J Biol Chem.

[28] Previs MJ (2023). Calcium activation through thick and thin?. J Gen Physiol.

[30] Spudich JA (2019). Three perspectives on the molecular basis of hypercontractility caused by hypertrophic cardiomyopathy mutations. Pflugers Arch.

[31] Day SM (2022). Myosin modulators: emerging approaches for the treatment of cardiomyopathies and heart failure. J Clin Invest.

[32] Maron BJ (1995). Prevalence of hypertrophic cardiomyopathy in a general population of young adults. Echocardiographic analysis of 4111 subjects in the CARDIA study. Coronary artery risk development in (young) adults. Circulation.

[33] Straceski AJ (1994). Functional analysis of myosin missense mutations in familial hypertrophic cardiomyopathy. Proc Natl Acad Sci U S A.

[34] Moore JR (2012). Understanding cardiomyopathy phenotypes based on the functional impact of mutations in the myosin motor. Circ Res.

[35] Kawana M (2022). Hypertrophic cardiomyopathy: mutations to mechanisms to therapies. Front Physiol.

[36] Wolf CM (2019). Hypertrophic cardiomyopathy: genetics and clinical perspectives. Cardiovasc Diagn Ther.

[37] Suay-Corredera C (2021). Protein haploinsufficiency drivers identify MYBPC3 variants that cause hypertrophic cardiomyopathy. J Biol Chem.

[38] Harvey PA, Leinwand LA (2011). The cell biology of disease: cellular mechanisms of cardiomyopathy. J Cell Biol.

[39] Green EM (2016). A small-molecule inhibitor of sarcomere contractility suppresses hypertrophic cardiomyopathy in mice. Science.

[40] Braunwald E (2023). Mavacamten: a first-in-class myosin inhibitor for obstructive hypertrophic cardiomyopathy. Eur Heart J.

[41] Rohde JA (2018). Mavacamten stabilizes an autoinhibited state of two-headed cardiac myosin. Proc Natl Acad Sci U S A.

[42] Willis MS (2009). Build it up-tear it down: protein quality control in the cardiac sarcomere. Cardiovasc Res.

[43] Martin TG, Kirk JA (2020). Under construction: the dynamic assembly, maintenance, and degradation of the cardiac sarcomere. J Mol Cell Cardiol.

[44] Wood NB (2022). Cardiac myosin filaments are maintained by stochastic protein replacement. Mol Cell Proteomics.

[45] Kelly CM (2023). Visualization of cardiac thick filament dynamics in ex vivo heart preparations. J Mol Cell Cardiol.

[46] Fleming PJ, Fleming KG (2018). HullRad: fast calculations of folded and disordered protein and nucleic acid hydrodynamic properties. Biophys J.

[47] Trybus JM (1982). A bent monomeric conformation of myosin from smooth muscle. Proc Natl Acad Sci U S A.

[48] Craig R (1983). Light-chain phosphorylation controls the conformation of vertebrate non-muscle and smooth muscle myosin molecules. Nature.

[49] Higuchi H, Ishiwata S (1985). Disassembly kinetics of thick filaments in rabbit skeletal muscle fibers. Effects of ionic strength, Ca2+ concentration, pH, temperature, and cross-bridges on the stability of thick filament structure. Biophys J.

[50] Irving M (2017). Regulation of contraction by the thick filaments in skeletal muscle. Biophys J.

[51] Marcucci L (2023). Muscle mechanics and thick filament activation: an emerging two-way interaction for the vertebrate striated muscle fine regulation. Int J Mol Sci.

[52] Josephs R, Harrington WF (1966). Studies on the formation and physical chemical properties of synthetic myosin filaments. Biochemistry.

[53] Saad AD (1986). Dynamic exchange of myosin molecules between thick filaments. Proc Natl Acad Sci U S A.

[54] Saad AD (1991). Visualization of myosin exchange between synthetic thick filaments. J Muscle Res Cell Motil.

[55] Katoh T (1998). Skeletal muscle myosin monomer in equilibrium with filaments forms a folded conformation. J Biol Chem.

[56] Takahashi T (1999). Conformations of vertebrate striated muscle myosin monomers in equilibrium with filaments. J Biochem.

[57] Wolny M (2013). Cardiomyopathy mutations in the tail of β-cardiac myosin modify the coiled-coil structure and affect integration into thick filaments in muscle sarcomeres in adult cardiomyocytes. J Biol Chem.

[58] Ojima K (2017). Myosin substitution rate is affected by the amount of cytosolic myosin in cultured muscle cells. Anim Sci J.

[59] Ichimura E (2022). Thick filament-associated myosin undergoes frequent replacement at the tip of the thick filament. FEBS Open Bio.

[60] Grillo MP (2019). In vitro and in vivo pharmacokinetic characterization of mavacamten, a first-in-class small molecule allosteric modulator of beta cardiac myosin. Xenobiotica.

[61] Caremani M (2021). Dependence of thick filament structure in relaxed mammalian skeletal muscle on temperature and interfilament spacing. J Gen Physiol.

[62] Milton DL (2011). Direct evidence for functional smooth muscle myosin II in the 10S self-inhibited monomeric conformation in airway smooth muscle cells. Proc Natl Acad Sci U S A.

[63] Beach JR (2014). Nonmuscle myosin II isoforms coassemble in living cells. Curr Biol.

[64] Scellini B (2021). Mavacamten has a differential impact on force generation in myofibrils from rabbit psoas and human cardiac muscle. J Gen Physiol.

[65] Toepfer CN (2019). SarcTrack. Circ Res.

[66] Bhagwan JR (2020). Isogenic models of hypertrophic cardiomyopathy unveil differential phenotypes and mechanism-driven therapeutics. J Mol Cell Cardiol.

[67] Schindelin J (2012). Fiji: an open-source platform for biological-image analysis. Nat Methods.

[68] O’Leary TS (2019). MYBPC3 truncation mutations enhance actomyosin contractile mechanics in human hypertrophic cardiomyopathy. J Mol Cell Cardiol.

