# Supplementary material for: Myosin folding boosts solubility in cardiac muscle sarcomeres
Source: JCI Insight. 2024 Mar 14;9(8):e178131. doi: 10.1172/jci.insight.178131 (PMC11141871; doi:10.1172/jci.insight.178131)
Supplement: Supplemental data [file jciinsight-9-178131-s197.pdf]

	Average	STDEV	Gene	Accession	Peptides
**Myosin Molecules**	**78.9%**	**4.0%**			
Myosin heavy chain	26.5%	2.6%	Myh7-Myh6	B2RQQ1; B2RY26	77
Myosin regulatory light chain 2, ventricular isoform	24.1%	2.8%	Myl2	P51667	19
Myosin light chain 3	27.7%	2.6%	Myl3	P09542	17
Myosin light chain 4	0.3%	0.4%	Myl4	Q9CZ19	7
Myosin, light polypeptide 7, regulatory	0.2%	0.3%	Myl7	Q5SVI8	8
Myosin light chain 1/3, skeletal muscle isoform	0.1%	0.0%	Myl1	P05977	2
Myosin-binding protein C, cardiac-type	2.7%	0.1%	Mybpc3	Q3UIK0	69
Tropomyosin 1, alpha, isoform CRA_l	3.5%	1.0%	Tpm1	Q545Y3	21
Putative uncharacterized protein	2.9%	1.5%	Gm10108	G3UWG1	7
Troponin T, cardiac muscle	1.9%	0.7%	Tnnt2	J3QQ13	9
Troponin C, slow skeletal and cardiac muscles	1.1%	0.6%	Tnnc1	P19123	6
Malate dehydrogenase, mitochondrial	0.9%	0.2%	Mdh2	P08249	15
Histone cluster 1, H1d	0.6%	0.2%	Hist1h1d	Q149Z9	2
Troponin I, cardiac 3	0.6%	0.0%	Tnni3	Q497F1	7
Histone H1.4	0.6%	0.2%	Hist1h1e	P43274	2
Alpha-globin	0.5%	0.1%	Hbat1	A8DUV1	2
Beta-globin	0.5%	0.1%	Hbbt1	D0U270	4
Histone H2B	0.5%	0.0%	LOC665622	Q921L4	4
Actin, alpha cardiac muscle 1	0.5%	0.4%	Actc1	P68033	4
Cysteine and glycine-rich protein 3	0.4%	0.1%	Csrp3	P50462	4
Histone H4	0.4%	0.1%	Hist2h4	B2RTM0	5
Histone H1.0	0.4%	0.1%	H1f0	P10922	4
Creatine kinase S-type, mitochondrial	0.4%	0.3%	Ckmt2	Q6P8J7	6
Myoglobin	0.3%	0.1%	Mb	P04247	5
Elongation factor 1-alpha 2	0.3%	0.1%	Eef1a2	P62631	2
Glyceraldehyde-3-phosphate dehydrogenase	0.2%	0.1%	Gapdh	A0A0A0MQF6	3
Beta-globin	0.2%	0.1%	Hbbt2	A8DV41	2
MCG140784	0.2%	0.4%	Try10	Q792Z1	2
ATP synthase subunit alpha, mitochondrial	0.2%	0.0%	Atp5a1	Q03265	8
PDZ and LIM domain protein 5	0.2%	0.0%	Pdlim5	E9Q8P5	4
Citrate synthase, mitochondrial	0.2%	0.1%	Cs	Q9CZU6	4
Very long-chain specific acyl-CoA dehydrogenase, mitochondrial	0.2%	0.2%	Acadvl	P50544	13
Actin, cytoplasmic 1	0.1%	0.0%	Actb	P60710	3
Medium-chain specific acyl-CoA dehydrogenase, mitochondrial	0.1%	0.1%	Acadm	P45952	5
Albumin 1	0.1%	0.1%	Alb	Q546G4	2
Trifunctional enzyme subunit beta, mitochondrial	0.1%	0.1%	Hadhb	Q99JY0	4
Myomesin-1	0.1%	0.0%	Myom1	Q62234	8
Keratin, type I cytoskeletal 10	0.1%	0.1%	Krt10	P02535	3
Putative uncharacterized protein	0.1%	0.0%	Acaa2	Q3UKH3	2
Cysteine-rich protein 2	0.1%	0.1%	Crip2	Q9DCT8	2
Trifunctional enzyme subunit alpha, mitochondrial	0.0%	0.0%	Hadha	Q8BMS1	3
Protein Ahnak	0.0%	0.0%	Ahnak	E9Q616	2
